# Modern Biophysics Redefines Our Understanding of Fungal Cell Wall Structure, Complexity, and Dynamics

**DOI:** 10.1128/mbio.01145-22

**Published:** 2022-05-31

**Authors:** Jean-Paul Latgé, Tuo Wang

**Affiliations:** a Institute of Molecular Biology and Biotechnology (FORTH), School of Medicine, University of Crete, Heraklion, Greece; b Department of Chemistry, Louisiana State University, Baton Rouge, Louisiana, USA; Duke University Medical Center

**Keywords:** *Aspergillus fumigatus*, cell wall, chitin, glucans, glycobiology, nuclear magnetic resonance

## Abstract

Even though the cell wall has been recognized as a crucial protective organelle for fungi, essential for its virulence and a unique emblem of this kingdom, biosynthesis of this organelle remains poorly understood. Our knowledge was based mainly in the past on the chemical analysis of cell wall mutants and on the biochemical study of a few synthases and transglycosidases. Recent developments in biophysical equipment and methods, such as solid-state nuclear magnetic resonance or cryo-electron microscopy, have promoted a better appreciation of the spatiotemporal dynamics of cell wall biosynthesis. The new information will be presented here with the cell wall of the human opportunistic pathogen Aspergillus fumigatus.

## PERSPECTIVE

The cell wall has been recognized as an essential protective organelle for fungi, especially for the terrestrial fungi which accumulate a large concentration of osmotically active molecules and harsh environmental stresses. In the 1950s, fungal cell studies were focused almost exclusively on a chemical analysis of this insoluble structure and on electron microscopy images which were revealing some structural specificity of the cell wall and the localization of the different polysaccharides as well as the heterogeneity between fungal species and fungal propagules (mycelium, asexual or sexual spores) (1–3). The recent introduction of biophysical approaches has changed our comprehension of the fungal cell wall. This essay which is based exclusively on Aspergillus fumigatus due to space limitation for an mBio perspective could be also extended to other filamentous fungi and yeasts.

## CELL WALL STRUCTURE IN 2021

The biochemical analytical strategies to study the fungal cell wall have not changed much over the years (4, 5). The cell wall is isolated from disrupted cells and solubilized with hot alkali or acids to separate amorphous and fibrillar polysaccharides. Solubilization of polysaccharides is a requirement prior to chemical analysis. Washing of nonconstitutive cell wall molecules and especially transitory glycoproteins is performed by boiling the disrupted cell wall in a mixture of a detergent, sodium dodecyl sulfate (SDS), and a reducing agent, such as mercaptoethanol or dithiothreitol (DTT), used to stabilize free sulfhydryl groups on proteins. In addition, this treatment would remove transglycosidases which are present in the cell wall and play a major role in the remodeling of cell wall polysaccharides synthesized by plasma membrane synthases (6). The use of gas chromatography-mass spectrometry after modification of the cell wall hexoses and hexosamines, the degradation of polysaccharides with specific (recombinant) glycosylhydrolases, and further separation of oligosaccharides by liquid chromatography and solution nuclear magnetic resonance (NMR) permit the identification of the different monomers of the cell wall and the linkages between the different sugars and oligosaccharides with homogenous repeating structures (4). In the case of A. fumigatus, the fibrillar alkali-insoluble core results from the covalent linkages of β-1,3-glucans, chitin, and galactomannan while the amorphous alkali-soluble material is composed mainly of α-1,3-glucans with some galactomannan (GM) and galactosaminogalactan (GAG) (7). The chemical data identify all the molecules and their covalent linkages, but the complex, three-dimensional organization of the polysaccharides remained unknown (8).

**FIG 1:**
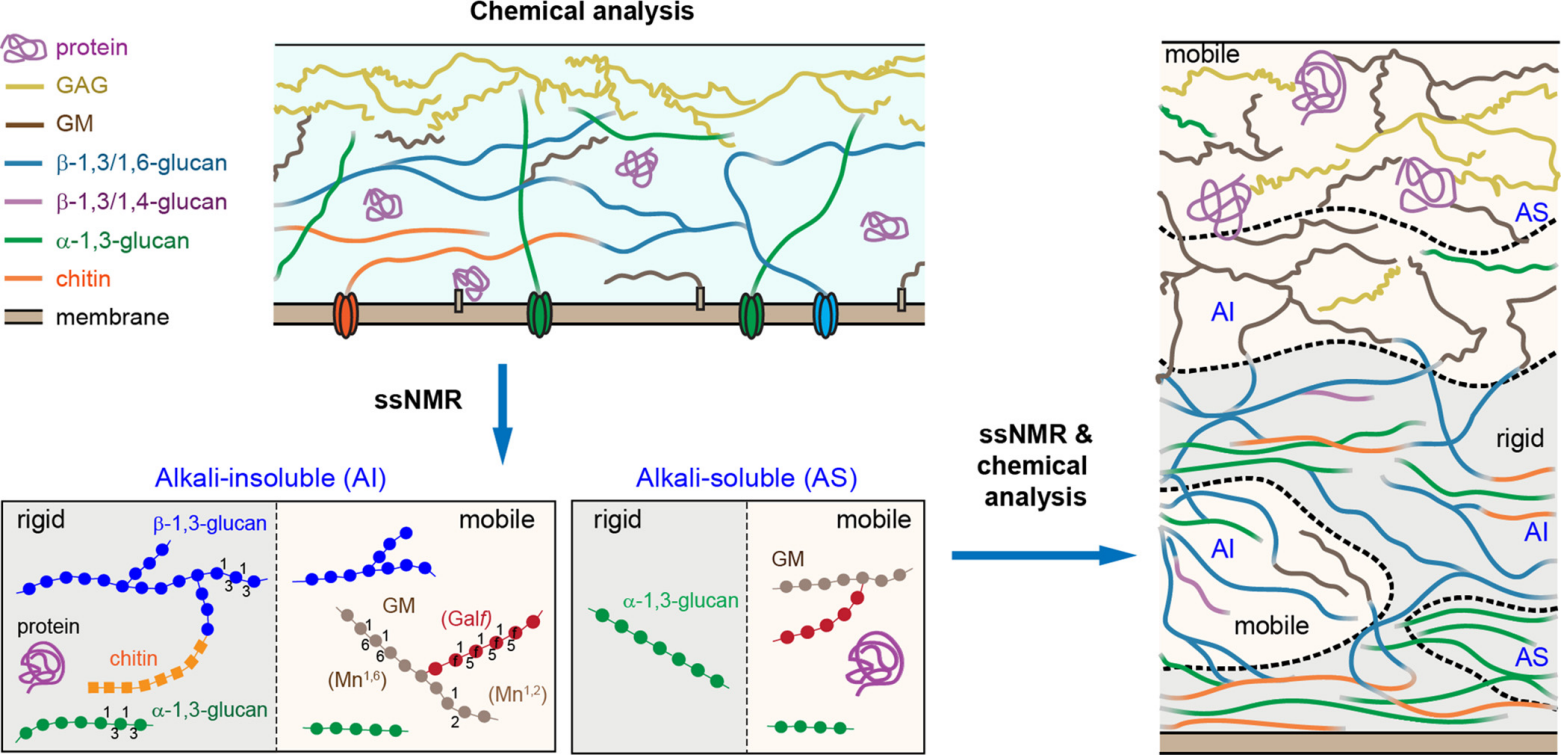
Organization of the hyphal cell walls obtained from chemical data. Modifications of the chemical structure after ssNMR studies showing the distribution of alkali-soluble (AS) and alkali-insoluble (AI) polymers in mobile and rigid domains. Current organization of cell wall molecules resulting from the combination of chemical and biophysical data. Figures are adapted from references 8 and 10 and are not to scale.

The recent application of solid-state nuclear magnetic resonance (ssNMR) spectroscopy has changed our view on the organization and dynamics of cell wall polymers which is different from the conclusions established biochemically over the past 5 decades (Fig. 1) (8–10).

ssNMR defines the dynamical and hydration characteristics of biopolymers which reflect the extent of molecular aggregation and water permeability (something that a chemical approach cannot perceive). These characteristics help to rationalize the structural organization of cell walls. Enhanced rigidity and hydrophobicity are typical indicators of large or ordered aggregates, such as chitin microfibrils. In contrast, molecules, such as β-1,3-glucans, spatially separated from these mechanical cores are typically mobile and hydrated. Chitin and β-glucans are joined together by covalent linkages, forming the mechanically rigid hot spots that are resistant to hot alkali treatment. The ssNMR analysis of the A. fumigatus cell wall, however, suggests that α-1,3-glucans are spatially packed with chitin and are likely distributed in a soft and hydrated hydrophobic matrix formed by diversely linked β-glucans. The α-1,3-glucan appears as the most rigid polysaccharide in A. fumigatus, and chitin is the second most rigid molecule. These α-1,3-glucans have a bimodal distribution of the motional dynamics, from which we can estimate that 70% to 90% of α-1,3-glucans interact with chitin to form a stiff and hydrophobic scaffold conferring rigidity to the cell wall while the other 10% to 30% interact with the mobile β-glucans. This estimation is supported by the many strong packing interactions (represented by carbon-to-carbon cross peaks) between chitin and α-1,3-glucan on the subnanometer length scale as well as their comparably low extent of water retention. This finding is very different from our previous chemical view where the most rigid molecule was chitin and α-1,3-glucan was only an amorphous independent polymer. At the same time, β-1,3-glucan has a moderately large number of cross peaks with both α-1,3-glucans and chitin but with much weaker intensities for the latter. Thus, chitin serves as a secondary anchor, following α-1,3-glucan, for β-1,3-glucan to link the rigid and mobile domains. These ssNMR studies have also confirmed that in fungi, like in plants, a single type of polysaccharides could possibly have mobile and rigid domains that fulfill different functions (10, 11). The occurrence of α-1,3-glucans in two distinct domains, namely, the outer surface and the inner rigid cores, demonstrates the structural and functional versatility of this molecule. However, until now it was not known if these molecules are part of the same polysaccharide or two dispersed “species” located in different layers of the cell wall. The development of new NMR instruments with up to 1,500 MHz (35.2 Tesla) (12) has improved the resolution of the cell wall structures in spite of its complexity (T. Wang, M. Dickwella Widanage, I. Hung, Z. Gan, T. Wang, unpublished). Currently, ssNMR shows that the α-1,3-glucan is physically associated with the chitin-β-glucan-GM core. However, the NMR data suggest that some β-glucans and α-1,3-glucans remain distant from chitin at the nanoscale; the former are mobile and alkali-insoluble while the latter are rigid but extractable. Therefore, there is no direct correlation between the chemical digestibility and the rigidity of a molecule. Moreover, the only polysaccharides which are essential as revealed by drug response and gene deletion are β-1,3-glucans and chitin, whereas α-1,3-glucans are easily dispensable in the cell wall. This finding means that essentiality and polymer organization are distinct.

## CELL WALL REMODELING IN RESPONSE TO STRESS

Gene deletion reshuffles the composition and spatial organization of polysaccharides with significant changes in their compositional dynamics, water accessibility, and mobility (10). Compensatory reactions in response to the absence of a cell wall component due to gene deletion have been observed, and data from chemical and enzymatic analyses (8) have been confirmed by ssNMR experiments (even though the conclusions of the chemical and biophysical approaches are not completely comparable) (10). Obviously, the lack of the major components leads to the most important cell wall changes. The absence of each of the two rigid polymers, α-1,3-glucan and chitin, led to an increase in the amount of alkali-insoluble fraction containing the fibrillar polysaccharides (13, 14). The lack of chitin and α-1,3-glucan was compensated by an increase in β-1,3-glucan as shown by biochemical assays. The removal of chitin, as revealed by ssNMR data, was also compensated by a significant increase in the α-1,3-glucan located in the rigid portion of the cell wall. In the sole mutant devoid of β-1,3-glucans, a compensatory increase of chitin and galactosaminogalactan and a significant decrease in galactomannan is seen (15). In contrast, the deletion of the galactosaminogalactan synthase gene leading to the disappearance of GAG, an extracellular polysaccharide present in a small amount, did not significantly modify the ratio of fibrillar and amorphous polysaccharides as distinguished using alkali treatment. Neither the mobility nor the rigidity of the major polysaccharides supports a structural role of GAG in the cell wall (16). The deletion of KTR mannosyltransferase genes leads to an increase in the amount of chitin (seen by chemical and ssNMR data) indicative of a concerted change of both rigid and mobile polymers (10, 17). Since a lack of a structural role is observed for galactofuranose or pyranose molecules, especially in galactomannan mutants which grow like their parental strain, it suggested that the mannan may have other structural roles that are not associated with GM which remain to be identified.

Data obtained by ssNMR or by chemical approaches in the analysis of the cell wall mutants confirmed that the composition and organization of polysaccharides have been fully reshuffled to better compensate for structural defects introduced by biosynthesis deficiencies or specific antifungal drugs or environmental changes. The joint biochemical, biophysical, and genetic analysis shows the high level of complexity in these compensatory mechanisms in response to cell wall stress and suggests that an interaction between the biosynthetic pathways is more complicated than previously appreciated. Accordingly, to date no rules can be established to predict these changes. In the cell wall mutants analyzed, the restructuring cell wall tends to increase the polymer rigidity but decrease the water retention in the mesh of the inner domain defining a new way for the fungal cell wall to fight against external insults. Accordingly, the balance between plasticity and rigidity maintained in the parental strain has been altered in the mutants, thus affecting cell growth. Here, again, no clear rules have been established (18).

## COMBINING BIOPHYSICAL AND CHEMICAL APPROACHES

The advantage of the ssNMR approach is that the cell wall is analyzed without any chemical perturbation; therefore, the physical and structural status of the cell wall is in its native configuration (19). ssNMR enables the detection of rigid and mobile molecules as defined by their native dynamics in the cell wall with no dependence on covalent linkage patterns or their susceptibility to chemical extraction. However, a limitation in the use of ssNMR results from an inadequate resolution due to the coexistence of many heterogeneous macromolecules in intact cell walls, some of which are present at low concentrations. To address this problem of sensitivity, the dynamic nuclear polarization (DNP) technique has been developed (20). A low concentration (e.g., 5 to 10 mM) of stable water-soluble biradicals are doped to biological samples so that we can transfer the spin polarization from the electrons of the biradicals to the nuclei (e.g., ^1^H, ^13^C and ^15^N) of the macromolecules to enhance NMR sensitivity by tens of fold. Using such development, ssNMR has identified the occurrence of eight chitin forms in A. fumigatus (21), whereas early data obtained by crystallography and NMR have identified only 3 types of chitin, as follows: α- and β-forms with adjacent chains packed in an antiparallel or parallel way and the γ-chitin, a putative form as a mixture of parallel and antiparallel packings. Interestingly, the covalent interactions with other polysaccharides in fungal cell walls do not seem to interfere with the establishment of the chitin forms. This unexpected level of structural polymorphism is potentially caused by the sophisticated pattern of hydrogen bonding through the N–H and C = O groups in chitin when multiple chains are put together outside the plasma membrane after the biosynthesis. It suggests that a subgroup of chitin allomorphs is responsible for forming rigid microfibrils, whereas the remainders retain considerable disorder due to unfavored conformations or unstable hydrogen-bonding patterns. It is unknown whether the observed polymorphism could be related to the biosynthetic function of diverse groups of chitin synthases involved in the biosynthesis of this polymer or to a different morphogenetic role of the different subspecies. In A. fumigatus, 8 chitin synthase genes (CHSs) have been found, and they belong to the three classes (I, II, and IV) that were also identified in yeasts but also have contributions from additional classes (III, V, VII, VI, or VIII) (14). It is clear that the deletion of each CHS does not lead to a class-specific phenotype. It will be now possible to investigate using single and multiple CHS mutants of A. fumigatus the genes responsible for each form and the chitin forms essential for correct morphogenesis, as a putative target for new antifungals.

ssNMR is ideal to obtain an overall cell wall structure but not sufficient to identify the covalent bridges occurring between cell wall components especially when present in low abundance. Accordingly, some of the branched β-1,3-glucans or deacetylated GlcNH_2_ present in a small amount in the cell wall are not seen by ssNMR. 1,8-Dihydroxynaphthalene (DHN) melanin has not been identified in A. fumigatus by ssNMR, but its deposition has been studied using Cryptococcus DOPA melanin. Small-angle X-ray scattering (SAXS) studies suggested that fungal melanin ghosts have a natural hollow structure with nonnegligible porosity and the capability to adsorb molecules. However, to date no exact ssNMR structure of a DHN melanin has been published, while this polymer has a key function in A. fumigatus pathobiology. More exploratory ssNMR studies are needed to address these underinvestigated aspects. In contrast, one of the interests of the use of ssNMR is its capacity to identify amino acids which gave strong signals in the cell wall. Accordingly, ssNMR was able to show the preponderance of valine especially in the alkali-insoluble fraction. The presence of rigid valine in the alkali-insoluble fraction suggests that it is not coming from intracellular proteins in transit to be secreted or cell wall-associated transglycosidases involved in cell wall remodeling, all cell wall-associated proteins which are normally removed by the alkali treatment (7, 10). These data suggested a covalent linkage of valine-containing peptides with the cell wall polysaccharides, but it remains to be identified if this amino acid has a structural role in the cell wall and which is its origin.

## MORE BIOPHYSICAL APPROACHES

Biophysical advances for the study of the fungal cell wall cannot be limited only to ssNMR. Atomic force microscopy was the first input of biophysical imaging on alive A. fumigatus (22). Recent developments in cryo-electron microscopy (cryoEM) with the availability of new imaging detectors and image analysis software have dramatically increased the acuity and analysis of images (23). Studies of the cell wall have followed the development of the microscope tools for years, and the introduction of these new imaging facilities should now be implemented for cell wall studies.

The synthesis of the major cell wall polysaccharides (β-1,3-glucan, chitin, or α-1,3-glucan) is undertaken by polysaccharide synthases which possess between 8 and 14 transmembrane domains. Moreover, if it has been recognized that the synthases are part of multiple protein complexes, their three-dimensional (3D) organization remains unknown. These proteins and encoding genes were identified only indirectly by genetic approaches leading to mutants which have specific alterations of their cell wall composition. Direct evidence of the activity is missing to date in the absence of the availability of any recombinant synthase since these proteins are of a large molecular size and have multiple transmembrane domains which have made their production impossible in a heterologous host.

**FIG 2:**
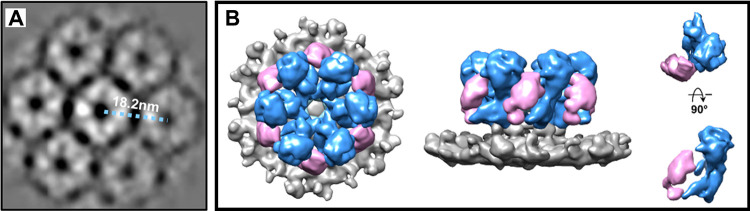
(A) Cryo-electron tomography two-dimensional (2D) average of the large ring-like structures in Candida glabrata. (B) Density map of the segmented putative β-1,3-glucan synthase. The figure is adapted from reference 25.

To understand the biophysical properties of the fungal cell wall and how it changes in response to antifungal drugs and environmental stresses, it will be important to have a better appreciation of the structure and organization of different key polysaccharide synthase complexes. CryoEM has become an essential technique to reveal the *in situ* organization and activity of transmembrane proteins (24). To date, only one publication has shown the organization of a fungal β-1,3-glucan synthase (25). The β-1,3-glucan synthase complex displays a hexameric complex (Fig. 2), with each monomeric unit being composed of a globular N-terminal domain and a larger central catalytic domain arranged as a top globular domain, a neck region, and a basal domain that connects the protein complex to the underlying membrane. This structure is partly reminiscent of the rosette structure of the plant cellulose synthase complex published recently (26).

Even though active recombinant fungal glucan synthases have not been produced, there are several experimental stratagems which should facilitate the use of cryoEM. The first one is the use of partially purified complexes using the product entrapment artifact (27) or regenerating protoplasts in the absence of an obscuring or blinding organized cell wall (25, 28). Alternatively, a liposome-like strategy could be used to introduce dye and nanobodies inside the cell. Chemical tools have emerged recently with chemical editable tags for nucleotide sugars that can be introduced into living cells after engineering the target enzymes in view to be directly used for superresolution microscopy (29, 30). Another alternative strategy is to examine homologous bacterial systems (belonging to the same GT2 family as the fungal polysaccharide synthases) which are known to synthesize glucans or chitin and can be expressed as active recombinant synthases (31–34). A drawback of the use of these bacterial systems is that the structure of the synthase, even though they lead to the same product, may be organized differently as seen with the plant and bacterial cellulose synthases (35). Similarly, the only fungal chitin synthase from Rhizopus oryzae, which has been expressed as an active protein in Escherichia coli, is a small CHSII protein whose structure is mostly hydrophilic, except for its carboxy end, and which may not be representative of all fungal chitin synthases (36). Another field that remains wide open and applicable for the cryoEM approach is the understanding of glycosylphosphatidylinositol (GPI)-anchored transglycosidases, which are responsible for the elongation and bridging between polysaccharides since these remodeling activities have been obtained *in vitro* with recombinant proteins (37). Moreover, the role of transitory or temporary components, such as putative extracellular vesicles that may interact with the structural cell wall, could be identified with superresolution microscopy approaches (28).

## CONCLUSION

Advances in the understanding of cell wall synthesis have been very slow in the last 50 years even though the cell wall is a unique drug target with molecules that are unique to fungi and essential to fungal life. The emergence of new biophysical approaches in combination with the genomic and chemical approaches has altered our view of the organization and dynamics of structural elements comprising the fungal cell wall. As enzymes important for cell function are among the most prominent targets for current and new antifungal drugs, understanding cell wall structure and dynamics is critical to better understanding their action. This perspective is focused almost exclusively on work on A. fumigatus. Additional works in the biophysical aspects of the cell wall (38) are going to be essential to make bridges on the cell walls of other fungi to support or undermine or homogenize the conclusions for the cell wall glycobiology.
